# Micro-Pattern of Graphene Oxide Films Using Metal Bonding

**DOI:** 10.3390/mi11040399

**Published:** 2020-04-10

**Authors:** Heba Abunahla, Nahla Alamoodi, Anas Alazzam, Baker Mohammad

**Affiliations:** 1 System-on-Chip Center, Electrical and Computer Engineering Department, Khalifa University of Science and Technology, Abu Dhabi 127788, UAE; heba.abunahla@ku.ac.ae (H.A.); baker.mohammad@ku.ac.ae (B.M.); 2 Research and Innovation Center on CO_2_ and H_2_ (RICH), Center of Catalysis and Separation (CeCaS), Chemical Engineering Department, Khalifa University of Science and Technology, Abu Dhabi 127788, UAE; 3 System-on-Chip Center, Mechanical Engineering Department, Khalifa University of Science and Technology, Abu Dhabi 127788, UAE

**Keywords:** metal, copper, graphene oxide, reduced graphene oxide, lithography, pattern, adhesion, dielectrophoresis

## Abstract

Recently, graphene has been explored in several research areas according to its outstanding combination of mechanical and electrical features. The ability to fabricate micro-patterns of graphene facilitates its integration in emerging technologies such as flexible electronics. This work reports a novel micro-pattern approach of graphene oxide (GO) film on a polymer substrate using metal bonding. It is shown that adding ethanol to the GO aqueous dispersion enhances substantially the uniformity of GO thin film deposition, which is a great asset for mass production. On the other hand, the presence of ethanol in the GO solution hinders the fabrication of patterned GO films using the standard lift-off process. To overcome this, the fabrication process provided in this work takes advantage of the chemical adhesion between the GO or reduced GO (rGO) and metal films. It is proved that the adhesion between the metal layer and GO or rGO is stronger than the adhesion between the latter and the polymer substrate (i.e., cyclic olefin copolymer used in this work). This causes the removal of the GO layer underneath the metal film during the lift-off process, leaving behind the desired GO or rGO micro-patterns. The feasibility and suitability of the proposed pattern technique is confirmed by fabricating the patterned electrodes inside a microfluidic device to manipulate living cells using dielectrophoresis. This work adds great value to micro-pattern GO and rGO thin films and has immense potential to achieve high yield production in emerging applications.

## 1. Introduction

Graphene, a monolayer of carbon, is widely believed to be the future of the technology advancement. The advantages of graphene material are almost unbeatable because of its excellent optical, electrical, and mechanical properties [1]. In spite of being the lightest existing material, graphene is the strongest compound known [2]. Scientists have carried out many researches related to the uses of graphene in many different fields such as electronics technology, environmental sciences, and various others [3,4,5]. Flexible electronics is one of the most promising applications of graphene because of its strong electrical properties, flexibility, and transparency. Graphene can revolutionize the next generation of chip technology, flexible displays, wearable devices, and communication data [6,7,8,9,10,11]. In the wireless field, prototypes of flexible communication antennas have been built, providing competitive solutions to flexible radio-frequency components [12].

Different methods have been used to prepare graphene [13]. Synthesizing graphene from graphene oxide (GO) is considered an effective approach to produce graphene in large scale, because of its low cost, high mass production, and decent solution processability [14]. GO is produced from graphite oxide which can be prepared in several ways. Being comprised of oxygen-containing groups, GO is described as an insulator rather than a conductor [15]. However, its conductivity can be increased by converting it into reduced graphene oxide (rGO). This can be achieved through chemical, thermal, or electrochemical reduction process [16], which is considered as a simple and cost-effective approach. 

Patterning GO or rGO is a high potential research field as it is essential to enable deploying graphene-related materials in various applications. Although the pattern scale is highly dependent on the targeted application of GO, micro-patterning holds high attention according to its broad spectrum of applications, especially for the transparent electrodes and flexible electronics [17]. Different GO pattern techniques are reported in the literature [18,19,20,21,22,23]. The functionality of the approach depends on the synthesis method of the GO and the target application. A recent work reports patterning solution-based GO on polymer flexible substrate using adapted lift-off method with plasma treatment [24]. The proposed approach is effective for fabricating devices with rGO micro-patterns produced by photolithography using aqueous solution of GO. Although water-based GO is a relatively stable material [25], its high surface tension makes it challenging to have a uniform GO films coated on polymer substrates, which is not optimal for high yield production, or when the uniformity of the films is a critical factor. The surface tension can be reduced by adding certain organic solvents to the aqueous GO solution, which improves the spread of GO on the substrate material and results in uniform GO thin films [26]. However, modifying the aqueous solution of GO with organic solvents might result in dissolving the photoresist layer that holds the desired micro-patterns, which in turn halts the accomplishment of the standard lift-off pattern technique [24].

The work proposed in this paper is a follow-up on the work reported in [24], aiming to improve the uniformity and yield of the target application. Here, we report a novel metal-based micro-pattern approach for modified aqueous GO solutions. The proposed technique can be used to pattern both GO and rGO film. However; the patterns provided in this work are performed on rGO film. The proposed pattern methodology takes advantage of the strong adhesion between the rGO film and metal copper (Cu) to enable patterning cyclic olefin copolymer (COC) substrates with modified GO solutions. It is observed that the bonding between a relatively thick deposited layer of Cu (500 nm and above) and a uniform rGO thin film is stronger than the bonds between the latter and the COC substrate. This results in peeling off the rGO film deposited on the substrate using the metal film upon immersion in acetone. Therefore, we propose to deposit rGO film, prepared from a modified aqueous GO solution, on COC substrate and pattern it using a photomask that contains the desired rGO micropatterns using photolithography. Next, by sputtering a Cu layer and dipping the wafer immediately in acetone, high stresses are created on the metal layer causing it to peel off from the substrate along with the rGO film that is in direct contact with it; hence leaving behind the rGO desired patterns protected by the photoresist layer. It is noteworthy that the adapted lift off approach proposed in [24] leads to a lower-cost fabrication compared to the pattern method provided in this paper. This is due to the extra metal deposition step used in this work and not needed in [24]. Thus, the work proposed in [24] is considered an effective approach if the uniformity of the GO film is not critical for the target application. On the other hand, the methodology provided in this work has immense potential to achieve high yield production in emerging applications. 

The rest of the paper is organized as follows. Section 2 details the used materials and the proposed patterning process, along with the fabrication of microchannel and bonding. In Section 3, a uniformity study of modified aqueous GO solutions is presented, and the use of the rGO micro-patterns for living cells manipulation under dielectrophoresis is demonstrated. Finally, conclusions about the methodology and the results achieved in this work are provided in Section 4.

## 2. Materials and Methods 

### 2.1. Micro-Patterning 

**Figure 1:**
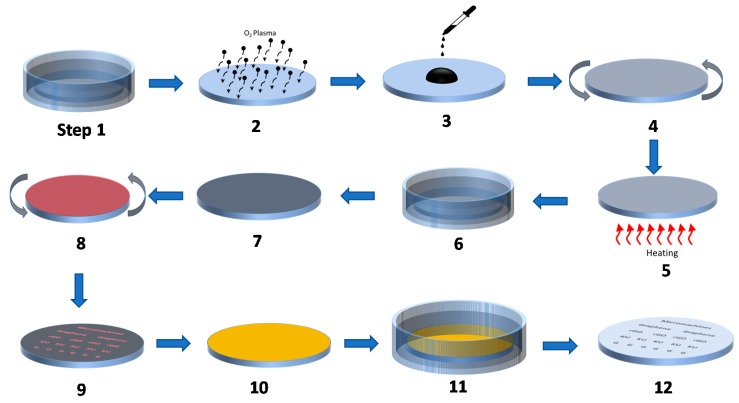
Schematic of the fabrication steps followed to achieve the proposed micro-pattern approach. Step **1**: Wafer cleaning. Step **2**: Plasma treatment. Step **3**,**4**: Graphene oxide (GO) deposition and spin coating. Step **5**: Baking. Step **6**,**7**: GO reduction in HI and then washing. Step **8**: Spin coating of photoresist. Step **9**: Photoresist patterning. Step **10**: Cu deposition. Step **11**,**12**: Photoresist lift-off in acetone to pattern the rGO layer.

The patterning process proposed in this work is performed on COC wafer because of the excellent adhesion between solution-based GO and COC substrate [24]. In addition, the COC substrate is considered a great asset for application related to flexible electronics technology. This enables building electronic circuits on flexible polymer substrates that can be deformed. Flexible electronics technology involves many emerging applications such as flexible memory, flexible sensors, and flexible batteries, which all are vital components of wearable and IoT devices [27]. The schematic diagram for the fabrication steps of rGO film pattern is shown in Figure 1. Step 1 involves cleaning the COC wafer by sonicating it in acetone, isopropanol, DI water, respectively. Later, the wafer is treated with plasma for one minute (step 2) to improve the adhesion between the GO flakes and the surface of the substrate. A layer of GO is deposited using spin coating and then the wafer is heated at 70 °C (steps 3–5). The GO solution used in this work is diluted by ethanol to alter its wettability and consequently the uniformity of the deposited GO layer on the substrate. In steps (6,7), the wafer is immersed in hydriodic acid to reduce GO to rGO. It is worth mentioning that the duration of this stage depends on the target conductivity of the rGO film, where higher conductivity requires longer immersion time. For instance, in resistive memory devices, high conductivity (reduction time ≥ 24 h) is needed if the pattern film is deployed as a conductive electrode. On the other hand, high resistivity (no reduction is needed) is desired if the film acts as the resistive switching material of the devices. For this work, an immersion time of 24 h is chosen to achieve an approximate conductivity of 45 S/cm. The lithography process is then accomplished by spin coating a thin layer of MICROPOSIT™ S1813™ positive photoresist on top of the rGO film (step 8). Next, the photoresist layer is patterned using a direct lithography system (Dilase 650 from KLOE) and then developed using MICROPOSIT™ MF-319 developer (step 9). At this stage, the desired rGO micro-patterns are protected by the photoresist layer. After that, the wafer is sputtered with copper (step 10) using a sputter coating system (Q300T T from Quorum Technologies). In step 11, the wafer is sonicated in acetone bath during which the full layer of copper is peeled off leaving behind the desired rGO patterns as presented in step 12. 

It is worth mentioning that in standard lift-off process, the GO is deposited on top of the patterned photoresist layer, which in the case of using modified aqueous GO, the photoresist layer can be dissolved resulting in the loss of the pattern. On the other hand, for the proposed approach, the process is reversed and the photoresist is patterned using a photomask with the desired GO pattern to serve as a protective layer over the GO-coated substrate, hence it is deposited after depositing the GO layer and heating the wafer. With this approach, the photoresist layer is not affected by the solvent used to prepare the modified aqueous GO.

### 2.2. Microchannel Fabrication and Bonding

The microfluidic device consists of a microchannel that is 2 mm wide and 50 µm deep and is made from polydimethylsiloxane (PDMS) bonded on a COC substrate with a patterned rGO film. The PDMS (Sylgard 184, Dow Corning, Midland, MI, USA) is fabricated using soft lithography process [28]. In brief, PDMS prepolymer is prepared by mixing it with the crosslinker in a 10:1 mass ratio. After degassing, the PDMS is poured into a silicon wafer consisting of SU-8 2050 (MicroChem Corp, Newton, MA, USA) microstructure of the channel prepared by photolithography. After baking for 2 h at 90 °C, the PDMS replica is peeled off and inlet and outlet ports are punctured. The PDMS microchannel is then pretreated with oxygen plasma at 690 mTorr before being silanized for one hour in a 6 wt% 3-(Trimethoxysilyl) propyl methacrylate (TMSPMA) (Sigma Aldrich, St. Louis, MO, USA) prepared in a 95 vol% ethanol solution. The silanization process assists in the bonding process between PDMS and the COC substrate. After patterning the COC wafer with rGO, both the PDMS microchannel and the COC wafer are plasma treated for three minutes. The microchannel is then aligned with the rGO pattern and pressed together with substrate gently. Finally, the device is kept for curing on a hotplate at 96 °C overnight. 

## 3. Results and Discussion 

### 3.1. Modified Aqueous GO Solution

**Figure 2:**
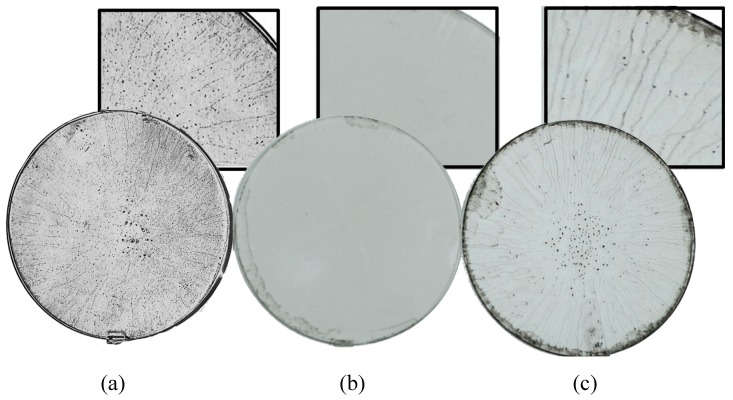
GO single layer spin coated on COC wafer using (**a**) aqueous dispersion of GO, (**b**) water–ethanol dispersion of GO, (**c**) water–acetone dispersion of GO. The concentration of the GO dispersions is 1 mg/mL.

As explained earlier, modified aqueous GO solution is used in this work to improve the uniformity of the deposited GO layer on COC substrates. Dispersions of GO in water are relatively stable because of the hydrogen bonds formed between the oxygen-containing functional groups in GO and water [25]. However, the deposition of thin films of aqueous GO on COC substrates for patterning rGO in microfluidic devices is challenging because of the nature of the substrate and the aqueous dispersions. The uniformity of the deposited film depends upon the effects of the polar and dispersive components of the surface energy of the GO dispersant. The surface energy ($\gamma_{S}$) of a pure component is defined as

(1) $$\gamma_{S}=\gamma^{p}+\gamma^{d}$$

where $\gamma^{p}$ is the polar component that accounts for the polar interactions including hydrogen bonding and permanent and induced dipoles, while $\gamma^{d}$ is the dispersive component that accounts for the instantaneous dipole moments [29]. Table 1 shows the surface energy and its components for different solvents and for the COC substrate. It can be observed that the polar component is dominant for the surface energy of water (51 mN/m), which requires substrates to have similar or higher polar components in order to wet them. In this context, since COC has a low polar energy component of 3.15 mN/m with a water contact angle of 101.2° [30], spin coating a COC wafer with an aqueous dispersion of GO results in a nonuniform thin film of GO. To decrease the surface tension of the GO dispersion, and hence improve its wetting property, a suitable organic solvent can be added to the GO aqueous solution. The choice of the organic solvent is based upon having a mild polar component, compared to water, to be compatible with the nonpolar COC. Though, the selected solvent should be polar enough to establish hydrogen bonds with water and GO. To illustrate the aforementioned concept, acetone and ethanol are selected to form modified aqueous solutions of GO using a 1:4 volume ratio of water-to-acetone and water-to-ethanol, respectively. The surface energies of acetone and ethanol are approximately the same, and their polar component is low in comparison to water. Figure 2 shows three COC wafers that are spin coated with aqueous dispersion of GO (Figure 2a), water–ethanol dispersion of GO (Figure 2b), and water–acetone dispersion of GO (Figure 2c). The three COC wafers are dipped in hydriodic acid after spin coating each of them with the associated GO solution. This step is performed to achieve clearer illustration of the results as the color of the GO layer darkens after reduction. Comparing the three wafers shown in Figure 2, the GO film deposited by the water–ethanol dispersion of GO appears to be highly uniform (Figure 2b). However, the aqueous dispersion of GO (Figure 2a) and the water-acetone dispersion of GO (Figure 2c) result in non-uniform deposition, with GO flakes concentrated at the center and striated radially outward. The non-uniformity of the aqueous GO solution is due to the hydrophobicity of the COC wafer, which explains the inability for water to wet it. In the case of the modified solutions, both acetone and ethanol completely wet the COC wafer. The difference in their ability to form a uniform GO film can be attributed to the intermolecular forces that are formed as they interact with water molecules and the functional groups grafted on the GO flakes. Both acetone and ethanol can form hydrogen bonds with water molecules. However, acetone can only form hydrogen bonds with GO in the presence of hydrogen-containing functional groups such as hydroxyl and carboxyl groups present in the flakes, this makes acetone a weak dispersant of GO. On the other hand, ethanol can form hydrogen bonds with GO because of the presence of the hydroxyl group in ethanol and the different oxygen-containing functional groups grafted on the GO flakes. This leads to a better dispersion of GO in the water–ethanol solution. The acetone- and ethanol-modified aqueous GO solution surface energies are estimated to be 27.04 [31] and 26 [32] mN/m, respectively. 

### 3.2. rGO Patterns

**Figure 3:**
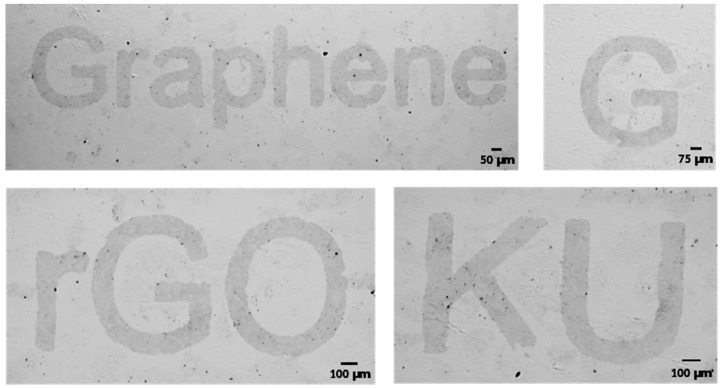
Optical microscopic images of the micro-patterns produced using the pattern methodology proposed in this work.

**Figure 4:**
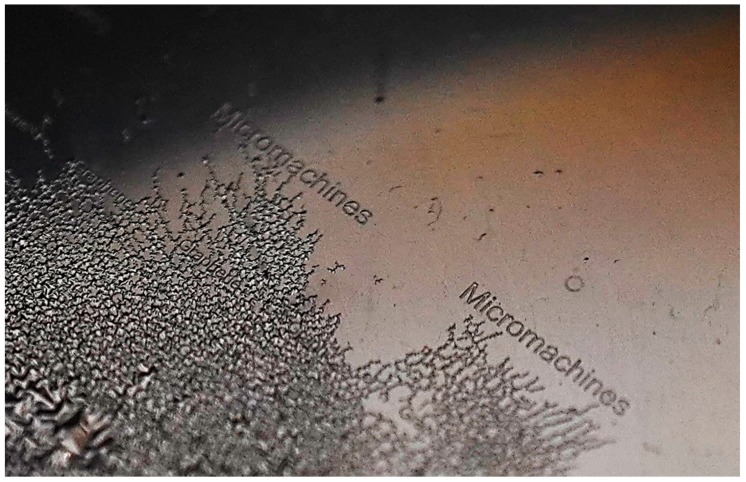
Optical image of the cracks generated directly after dipping the wafer in acetone, after copper deposition. The full Cu film peels off from the substrate and the rGO under the Cu film is detached with it from the COC substrate.

In this section, we present high resolution and precision micro-patterns of rGO produced using the methodology detailed in Section 2. Figure 3 shows the optical microscopic images of rGO patterns of line width ranging from 50–100 µm. These results prove the feasibility, repeatability, and scalability of the proposed approach. The images show that the patterned rGO film is uniform and have neat edges. Two key factors facilitate accomplishing the fabrication process proposed in this work. First, the bond strength of Cu and rGO films is considered an essential aspect in this approach. The second factor is the stresses experienced by the sputtered Cu layer upon immersion in a solvent (i.e., acetone) immediately after sputtering. The bonds formed between Cu and rGO are strong compared to rGO and COC, which causes the rGO film attached to the Cu layer to be removed upon peeling off the metal. The rGO flakes are physically adsorbed on the surface of the COC wafer during deposition, however the Cu sputtering over the GO coated COC causes a chemical bonding of Cu to oxygen containing functional groups in the rGO film. The mechanism of this bonding was previously studied for sputtering different metals on graphene and GO [33]. It was reported that upon sputtering of Cu, Cu^2+^ ions will be readily exchanged with H^+^ existing at COOH groups found at the GO/rGO surface, and consequently forming Cu(COO)_2_. In another study, metal ions were found to bond with graphene after forming defects on the graphene surface due to sputtering [34]. Their findings were validated by showing that no defects were formed by other metal deposition methods which result in the removal of the metal without the graphene upon immersion in acid. The intrinsic force in the Cu film depends on the substrate temperature during the deposition and the difference in the thermal expansions for the substrate and deposited copper film. In this work, the thick film of Cu is deposited on top of the rGO film at high sputtering pressure (1 Pa) and high deposition rate (1 nm/s). Performing the sputtering at these conditions and having the thermal expansion of COC almost four time the one of Cu (17 × 10^−6^ °C^−1^ for Cu and 60 × 10^−6^ °C^−1^ for COC) increase the intrinsic force per unit width in the Cu film [35]. The development of stresses in the Cu film results in high-density crack growth once the substrate is immersed in acetone as shown in Figure 4. As the COC-rGO bond is weaker than the rGO-Cu bond, the Cu film peels off from the substrate and the rGO under the Cu film is detached with it from the COC substrate. Acids were not used to dissolve the Cu film since it removes the Cu film without the rGO beneath it. 

### 3.3. Living Cells Manipulation 

**Figure 5:**
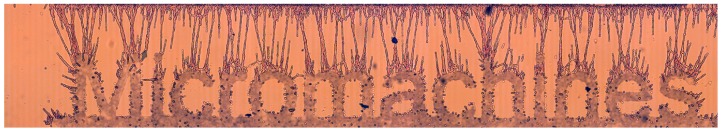
A microscopic image of pDEP response of RBCs under an AC signal of 5 Vpp at 1 MHz.

The fabrication process proposed in this work is utilized to pattern rGO film to make electrodes inside a microfluidic device and use them to manipulate living cells. The rGO film is patterned to create two electrodes which are then connected to an AC signal to generate inhomogeneous electric field inside the microdevice. One electrode is patterned to make the word “micromachines” and the other one is made as a simple straight line. The generated inhomogeneous electric field induces lateral motion on cells because of dielectrophoresis (DEP). The DEP is defined as the motion of neutral and polarizable particles or cells suspended in a conductive medium under the effect of inhomogeneous electric field [36,37,38]. It is a label-free manipulation method with low impact on the cellular integrity [38]. The movement of cells under DEP will be toward the high gradient of the electric field if the polarizability of the cells is higher than the one of the carrying medium. This phenomenon is called positive DEP (pDEP). Alternatively, negative DEP (nDEP) occurs when the cells move toward the low gradient of electric field, away from the electrodes, as a result of having their polarizability less than the one of the carrying mediums [39,40]. The magnitude of the DEP force depends on several parameters discussed previously in several publications [37,38,39,40,41]. However, human red blood cells (RBCs) experience pDEP at AC frequency in the range of 1–3 MHz [6]. RBCs from a healthy donor are suspended in an isotonic sucrose-dextrose medium and then introduced to the microfluidic device using a syringe pump. The channel fabrication, medium and samples preparations, are detailed in [40,41,42]. Figure 5 shows the pDEP response of RBCs under an AC signal of 5 Vpp at 1 MHz. The accumulation of cells between the straight upper electrode and the “micromachines” electrode is clearly visible. Cells accumulated in the regions of highest electric field strength that occur between the upper straight electrode and the tips of the “micromachines” electrode. This confirms the suitability of the patterned rGO film to be used as a conductive film (electrodes). 

## 4. Conclusions

This work presented novel fabrication process to achieve uniform micro-patterns of rGO on COC substrate. This was facilitated by spin coating COC with water–ethanol dispersion of GO. The results showed substantial improvement in the deposition uniformity among the rGO films because of the high wettability of the used solution. Thin films prepared by aqueous GO modified by organic solvents is difficult to be patterned using standard lift-off process reported in the literature as the used solvents might dissolve the photoresist layer and consequently abolish the patterns. In this work, the strong adhesion between rGO and metal Cu compared to the bonds between rGO and polymer substrates was utilized. The possibility of patterning rGO films by peeling off the areas directly attached to Cu (not protected with photoresist) was demonstrated in this paper. The repeatability of the methodology was confirmed by fabricating different patterns with different sizes. Moreover, the feasibility of patterning electrodes for blood cell manipulation in microfluidic device was successfully achieved. It is worth mentioning that the proposed micro-pattern method can also work with other metal films and the use of Cu was a representative case. This can be attained via careful optimization of the metal deposition process to achieve the optimal metal thickness needed to pattern the rGO film.

## Figures and Tables

**Table 1:** Surface energy of cyclic olefin copolymer (COC) and different solvents in mN/m.

Component	$\gamma_{S}$	$\gamma^{p}$	$\gamma^{d}$
Deionized water	72.80	51.00	21.80
Acetone	23.30	16.50	6.80
Ethanol	23.70	4.40	19.30
COC	45.65	3.15	42.50

## References

[1] Ki Hong S., Eun Kim J., Kim S.O., Jin Cho B. (2011). Analysis on switching mechanism of graphene oxide resistive memory device. J. Appl. Phys..

[2] Mikhailov S. (2011). Physics and Applications of Graphene: Experiments.

[3] Bhat U., Meti S. (2020). Graphene-Based ZnO Nanocomposites for Supercapacitor Applications. Graphene Energy Storage Mater. Supercapacitors.

[4] Alazzam A., Alamoodi N., Abutayeh M., Stiharu I., Nerguizian V. (2019). Fabrication of porous gold film using graphene oxide as a sacrificial layer. Materials.

[5] Alazzam A., Alamoodi N. (2020). Microfluidic Devices with Patterned Wettability Using Graphene Oxide for Continuous Liquid-Liquid Two-Phase Separation. ACS Appl. Nano Mater..

[6] Stanford M.G., Zhang C., Fowlkes J.D., Hoffman A., Ivanov I.N., Rack P.D., Tour J.M. (2020). High-Resolution Laser-Induced Graphene. Flexible Electronics beyond the Visible Limit. ACS Appl. Mater. Interfaces.

[7] You R., Liu Y.Q., Hao Y.L., Han D.D., Zhang Y.L., You Z. (2019). Laser fabrication of graphene-based flexible electronics. Adv. Mater..

[8] Uz M., Jackson K., Donta M.S., Jung J., Lentner M.T., Hondred J.A., Claussen J.C., Mallapragada S.K. (2019). Fabrication of High-resolution Graphene-based Flexible electronics via polymer Casting. Sci. Rep..

[9] Peng P., Li L., He P., Zhu Y., Fu J., Huang Y., Guo W. (2019). One-step selective laser patterning of copper/graphene flexible electrodes. Nanotechnology.

[10] Sahoo M., Wang J.C., Nishina Y., Liu Z., Bow J.S., Lai C.S. (2020). Robust sandwiched fluorinated graphene for highly reliable flexible electronics. Appl. Surf. Sci..

[11] Ballesio A., Parmeggiani M., Verna A., Frascella F., Cocuzza M., Pirri C.F., Marasso S.L. (2019). A novel hot embossing Graphene transfer process for flexible electronics. Microelectron. Eng..

[12] Filter R., Farhat M., Steglich M., Alaee R., Rockstuhl C., Lederer F. (2013). Tunable graphene antennas for selective enhancement of THz-emission. Opt. Express.

[13] Choi W., Lahiri I., Seelaboyina R., Kang Y.S. (2010). Synthesis of graphene and its applications: A review. Crit. Rev. Solid State Mater. Sci..

[14] Xue Y., Zhu L., Chen H., Qu J., Dai L. (2015). Multiscale patterning of graphene oxide and reduced graphene oxide for flexible supercapacitors. Carbon.

[15] Dimiev A.M., Eigler S. (2016). Graphene Oxide: Fundamentals and Applications.

[16] Pei S., Cheng H.M. (2012). The reduction of graphene oxide. Carbon.

[17] Kim T., Kim H., Kwon S.W., Kim Y., Park W.K., Yoon D.H., Jang A.-R., Shin H.S., Suh K.S., Yang W.S. (2012). Large-scale graphene micropatterns via self-assembly-mediated process for flexible device application. Nano Lett..

[18] Le L.T., Ervin M.H., Qiu H., Fuchs B.E., Lee W.Y. (2011). Graphene supercapacitor electrodes fabricated by inkjet printing and thermal reduction of graphene oxide. Electrochem. Commun..

[19] Guo Y., Di C.A., Liu H., Zheng J., Zhang L., Yu G., Liu Y. (2010). General route toward patterning of graphene oxide by a combination of wettability modulation and spin-coating. ACS Nano.

[20] Pang S., Tsao H.N., Feng X., Müllen K. (2009). Patterned graphene electrodes from solution-processed graphite oxide films for organic field-effect transistors. Adv. Mater..

[21] Dua V., Surwade S.P., Ammu S., Agnihotra S.R., Jain S., Roberts K.E., Park S., Ruoff R.S., Manohar S.K. (2010). All-organic vapor sensor using inkjet-printed reduced graphene oxide. Angew. Chem. Int. Ed..

[22] Ryu Y.K., Garcia R. (2017). Advanced oxidation scanning probe lithography. Nanotechnology.

[23] Kurra N., Reifenberger R.G., Kulkarni G.U. (2014). Nanocarbon-scanning probe microscopy synergy: Fundamental aspects to nanoscale devices. ACS Appl. Mater. Interfaces.

[24] Alazzam A. (2020). Solution-based, flexible, and transparent patterned reduced graphene oxide electrodes for lab-on-chip applications. Nanotechnology.

[25] Sun P., Zhu M., Wang K., Zhong M., Wei J., Wu D., Zhu H. (2013). Selective ion penetration of graphene oxide membranes. ACS Nano.

[26] Konios D., Stylianakis M.M., Stratakis E., Kymakis E. (2014). Dispersion behaviour of graphene oxide and reduced graphene oxide. J. Colloid Interface Sci..

[27] Aleksandrova D. Five Benefits of Flexible Electronics for Displays and Sensors. https://www.flexenable.com/blog/five-benefits-of-flexible-electronics-for-displays-and-sensors/.

[28] Xia Y., Whitesides G.M. (1998). Soft lithography. Annu. Rev. Mater. Sci..

[29] Hwang S.J., Tseng M.C., Shu J.R., Yu H.H. (2008). Surface modification of cyclic olefin copolymer substrate by oxygen plasma treatment. Surf. Coat. Technol..

[30] Cortese B., Mowlem M.C., Morgan H. (2011). Characterisation of an irreversible bonding process for COC–COC and COC–PDMS–COC sandwich structures and application to microvalves. Sens. Actuators B Chem..

[31] Enders S., Kahl H., Winkelmann J. (2007). Surface tension of the ternary system water + acetone + toluene. J. Chem. Eng. Data.

[32] Khattab I.S., Bandarkar F., Fakhree M.A.A., Jouyban A. (2012). Density, viscosity, and surface tension of water + ethanol mixtures from 293 to 323 K. Korean J. Chem. Eng..

[33] Ogata C., Koinuma M., Hatakeyama K., Tateishi H., Asrori M.Z., Taniguchi T., Funatsu A., Matsumoto Y. (2014). Metal permeation into multi-layered graphene oxide. Sci. Rep..

[34] Dimiev A., Kosynkin D.V., Sinitskii A., Slesarev A., Sun Z., Tour J.M. (2011). Layer-by-layer removal of graphene for device patterning. Science.

[35] Pletea M., Brückner W., Wendrock H., Kaltofen R. (2005). Stress evolution during and after sputter deposition of Cu thin films onto Si (100) substrates under various sputtering pressures. J. Appl. Phys..

[36] Stiharu I., Alazzam A., Nerguizian V., Roman D. (2015). Single living cell manipulation and identification using microsystems technologies. Microsyst. Nanoeng..

[37] Waheed W., Alazzam A., Abu-Nada E., Khashan S., Abutayeh M. (2018). A microfluidics device for 3D switching of microparticles using dielectrophoresis. J. Electrost..

[38] Waheed W., Alazzam A., Mathew B., Christoforou N., Abu-Nada E. (2018). Lateral fluid flow fractionation using dielectrophoresis (LFFF-DEP) for size-independent, label-free isolation of circulating tumor cells. J. Chromatogr. B.

[39] Nerguizian V., Stiharu I., Al-Azzam N., Yassine-Diab B., Alazzam A. (2019). The effect of dielectrophoresis on living cells: Crossover frequencies and deregulation in gene expression. Analyst.

[40] Mathew B., Alazzam A., Khashan S., Abutayeh M. (2017). Lab-on-chip for liquid biopsy (LoC-LB) based on dielectrophoresis. Talanta.

[41] Alazzam A., Stiharu I., Bhat R., Meguerditchian A.N. (2011). Interdigitated comb-like electrodes for continuous separation of malignant cells from blood using dielectrophoresis. Electrophoresis.

[42] Alhammadi F., Waheed W., El-Khasawneh B., Alazzam A. (2018). Continuous-Flow Cell Dipping and Medium Exchange in a Microdevice using Dielectrophoresis. Micromachines.

